# Comorbidity in myasthenia gravis: multicentric, hospital-based, and controlled study of 178 Italian patients

**DOI:** 10.1007/s10072-024-07368-0

**Published:** 2024-02-22

**Authors:** Vincenzo Di Stefano, Salvatore Iacono, Massimiliano Militello, Olga Leone, Marianna Gabriella Rispoli, Laura Ferri, Paola Ajdinaj, Placido Lanza, Antonino Lupica, Grazia Crescimanno, Roberto Monastero, Antonio Di Muzio, Filippo Brighina

**Affiliations:** 1 Department of Biomedicine, Neuroscience, and Advanced Diagnostic (BIND), University of Palermo, Palermo, Italy; 2 Department of Neuroscience, Imaging and Clinical Sciences, “G. D’Annunzio” University, Chieti, Italy; 3 Institute for Biomedical Research and Innovation, National Research Council of Italy, Palermo, Italy

**Keywords:** Myasthenia gravis, Comorbidity, Thyroid disorders, Hypertension, Osteoporosis, Autoimmune diseases

## Abstract

**Background:**

Myasthenia gravis (MG) is an autoimmune disorder with fluctuating weakness that causes significant disability and morbidity. Comorbidities may influence the course of MG, particularly in specific subgroups. The aim of this study is to investigate the frequency of comorbidities in MG patients compared to healthy controls (HC) and to evaluate their distribution according to age at disease onset, sex, and disease severity.

**Methods:**

MG patients attending the University Hospital “Paolo Giaccone” in Palermo and “SS Annunziata” Hospital in Chieti were enrolled; HC were enrolled from the general population. Non-parametric statistics and logistic regression were used to assess the association of specific comorbidities according to age at disease onset, sex, disease subtypes, and severity of the disease.

**Results:**

A total of 356 subjects were included in the study: 178 MG patients (46% F; median age 60 years [51–71]) and 178 sex- and age-matched HC (46% F, median age 59 years [50–66]). Overall, 86% of MG patients and 76% of HC suffered from comorbidities, and MG patients had a higher number of comorbidities compared to HC. Patients with late-onset suffered from more comorbidities than those with early-onset MG. Hypertension was more common in male patients with MG, while thymic hyperplasia, osteoporosis, and autoimmune diseases were more common in females. Respiratory disorders and thymoma were more common in patients with more severe disease (*p* < 0.05 for all comparisons).

**Conclusion:**

MG patients, particularly those with late onset, showed a higher prevalence of comorbidities than HC. Assessment of comorbidities in MG is an essential issue to identify the appropriate treatment and achieve the best management.

**Supplementary Information:**

The online version contains supplementary material available at 10.1007/s10072-024-07368-0.

## Introduction

Myasthenia gravis (MG) is an autoimmune disorder with fluctuating weakness and specific autoantibodies to the acetylcholine receptor (AChR), muscle-specific kinase (MuSK), or low-density lipoprotein receptor–related protein 4 (LRP4), titin, or agrin at the neuromuscular junction [1, 2]. MG is classified into different subtypes depending on the age at disease onset (“early onset,” EO before 50 years; “late onset,” LO between 50 and 65 years; “very late onset,” VLO after 65 years) [3], the distribution of weakness (“ocular,” OMG; “generalized,” GMG), the presence of thymoma, and the response to treatments (classical forms versus refractory MG) [4]. Although MG is considered a rare disease, its impact is significant for patients and their caregivers, causing significant disability and morbidity in affected patients [5]. In fact, MG patients have a fluctuating course of the disease with silent periods alternating with exacerbations [5]. Of note, the COVID-19 pandemic demonstrated the role of infections and triggers in MG exacerbation [6, 7]. Comorbidity can significantly influence the quality of life, life expectancy, and treatment choices in MG[8–12]. For example, B-cell depletion therapies are not indicated in patients with pre-existing hematological disease [13], while azathioprine cannot be used in the case of liver failure [14], and steroids might precipitate hypertension spikes, bone fractures, and diabetes [15]. For this reason, the development of specialized centers for the care of MG patients, as well as the use of new effective treatments, has recently become an essential standard of care for improving the survival of MG patients, reducing the overall complication rate [11]. Comorbidities are described in 74–95% of patients, and, interestingly, their presence is usually associated with more frequent hospitalizations and myasthenic crises[8, 10, 11, 16, 17]. Furthermore, sex has been shown to play a role in both epidemiological and pathophysiological aspects of comorbidity in MG [18]. Also, the role of comorbidity has been recently debated for LOMG and VLOMG [10, 16, 19]. In this scenario, the prolonged survival of MG patients and the safety issues from immunosuppressive therapies have both emphasized the role of comorbidity in MG [11]. However, only a few studies have evaluated the role of comorbidities in MG [8–11, 16, 17]. In the present study, the role of comorbidities in MG was evaluated; such data will be useful for estimating the impact of comorbidities on the course of the disease and the management of its complications.

## Materials and methods

### Study design and aims

We conducted a cross-sectional, retrospective, and multicentric, hospital-based, study involving the Neurological Clinics of “Policlinico Paolo Giaccone” University Hospital of Palermo and “SS Annunziata” Hospital od Chieti. The primary aim of this study was to assess the prevalence of autoimmune and non-autoimmune comorbidities in people with MG and those without a diagnosis of MG (i.e., healthy controls, HC), to explore whether there is an association between comorbidities and MG. The secondary aims were as follows: (1) to investigate the association between comorbidities and age of MG onset, (2) to explore the distribution of comorbidities in people with MG according to sex, (3) to investigate the association between comorbidities and generalized MG (GMG), (4) to investigate the association between comorbidities and MGFA class, (5) to investigate the association between comorbidities and MG treatments.

### Myasthenia gravis subjects

Patients with a definite diagnosis of MG admitted to the Neurological Clinics of “Policlinico Paolo Giaccone” University Hospital of Palermo and “SS Annunziata” Hospital od Chieti in the period between June 2021 and June 2022 were included. Inclusion criteria for patients with MG were a diagnosis of MG according to existing recommendations [14, 20], a regular 6-month follow-up, and a signed written informed consent form to participate in the study. Disease severity was assessed using the Myasthenia Gravis Foundation of America (MGFA) classification [21]. The medical history of all the MG participants was collected through the retrospective evaluation of medical records. In addition, the medical history was confirmed and/or updated for each participant at scheduled follow-up visits carried out between June 2021 and July 2022. The enrolled MG patients were classified according to the clinical phenotype (i.e., ocular MG [OMG], generalized MG [GMG]) and age at MG onset (i.e., early onset [age < 50 years]; late onset [age ≥ 50 years]) [3].

### Not-myasthenic subjects and matching criteria

HC were collected among the people living in the areas of Sicily and Abruzzo, Italy, through a web-based questionnaire processed in Google Forms (Google LLC, Menlo Park, CA, USA). The questionnaire was self-administered and contained 29 mandatory questions, 17 of which included a double choice (i.e., yes or no) concerning the informed consent form to participate in the study, sex, and the presence or absence of the listed comorbidities, while the remaining 12 ones allowed an open-ended response in which participants were invited to specify age in years and the type of comorbidity if it had been previously selected. The invitation to participate in this survey was widespread through web mail and social media during the period from May to September 2021. The English survey questionnaire is attached to the supplementary materials (Supplementary File 1). We established a case/control ratio of 1:1 using sex (i.e., female/male ratio of 1:1 between cases and controls) and age (i.e., ± 5 years) as matching criteria.

### Laboratory testing

Testing for autoantibodies (i.e., anti-AChR, anti-MuSK) was performed by the radioimmunoassay method using a radio-receptor assay kit. Specifically, anti-AChR and anti-MuSK autoantibodies were measured by two different commercially available ELISA methods for detecting AChR (RSR Ltd., Cardiff, UK) and MuSK autoantibodies (IBL International GmbH, Männedorf, Germany). The results were reported as positive for AChR if > 0.50 nmol/l, and positive for anti-MuSK if > 0.40 U/ml [22].

All the MG patients underwent thyroid hormones and thyroid stimulating hormone serum testing, and anti-nucleus antibodies testing at least once time during the entire follow-up. In addition, MG patients routinely underwent laboratory testing for complete blood count, creatinine, blood urea nitrogen, transaminases, gamma-glutamyl transferase, and total and fractionated bilirubin allowing us to assess pathological changes.

### Comorbidity collection

Information pertaining to comorbidities was collected using medical reports from previous hospitalizations in ordinary or day hospital settings, as well as reports from outpatient specialist visits. Among medical records, all diagnoses were recorded based on the patient self-reported history, instrumental examinations performed, and the official reports of the consulting medical specialists. These data concerning comorbidities were also updated for each patient at the scheduled follow-up visit by examining new or unknown medical reports during the entire study period. All diagnoses collected were coded according to ICD-9 in accordance with the diagnostic codes of the Italian National Health System. Furthermore, all the MG participants underwent computed tomography or magnetic resonance imaging of the mediastinum which was performed as a part of a diagnostic work-up to assess the presence of thymic hyperplasia and/or thymoma. The following comorbidities were collected: (1) hypertension (i.e., history of documented hypertension; blood pressure ≥ 140/90 mmHg; use of antihypertensive drugs), (2) diabetes mellitus (i.e., history of documented diabetes; fasting serum glucose ≥ 126 mg/dl in two determination; glycated hemoglobin ≥ 6.5%; use of antidiabetic drugs), (3) hypercholesterolemia (i.e., history of documented hypercholesterolemia; serum cholesterol ≥ 200 mg/dl; low-density lipoprotein ≥ 100 mg/dl; use of statins), (4) osteoporosis (i.e., evidenced on bone densitometry), (5) thyroid diseases (i.e., nodules, goiter, Hashimoto’s thyroiditis, Graves’ disease), (6) kidney diseases (i.e., chronic renal failure, renal cyst, renal stones, autoimmune glomerulonephritis), (7) cardiovascular diseases (i.e., ischemic cardiopathy, arrythmias, ventricular hypertrophy, cardiac failure, valvulopathies, atherosclerosis, aneurysm), (8) ocular diseases (i.e., cataract, glaucoma, retinopathy), (9) gastrointestinal diseases (i.e., gastroesophageal reflux disease [GERD], gastritis, gallstones, liver diseases, inflammatory bowel diseases [IBD]), (10) hematological diseases (i.e., anemia, other cytopenia, monoclonal gammopathy of undetermined significance [MGUS], hypogammaglobulinemia, coagulopathies), (11) non-thymic tumors (i.e., uterine, breast, prostate, urinary, cutaneous, respiratory, gastrointestinal, central nervous system, glands, lymphoma), (12) respiratory diseases (i.e., chronic obstructive pulmonary diseases [COPD], asthma, obstructive sleep apnoea syndrome [OSAS], interstitial lung disease), (13) neurological diseases (history of stroke or transient ischemic attack [TIA], migraine, epilepsy, Parkinson’s diseases and other parkinsonism, neuropathies, motor neuron diseases, Stiff-person syndrome, fibromyalgia, radiculopathy), (14) psychiatric diseases (i.e., anxiety, depression, psychosis), (15) systemic autoimmune diseases (i.e., rheumatoid arthritis, undifferentiated connectivitis, Sjogren syndrome, erythematous systemic lupus, vasculitis, psoriasis, polymyalgia). Organ-specific autoimmune comorbidities (e.g., thyroid autoimmune diseases, chronic inflammatory polyneuropathy [CIDP], IBD) have been considered separately from the other non-autoimmune organ-specific comorbidity, and they were grouped together with systemic autoimmune comorbidities into a single category named “Autoimmune comorbidity”; thus, organ-specific autoimmune comorbidities have been counted only once in the statistical analyses.

HC responses were anonymous and confidential in accordance with Google’s privacy policy (https://policies.google.com/privacy?hl=en). No contact information was collected. The web-based questionnaire required participants to obtain informed consent. If consent was denied, the questionnaire ended automatically, and the response was not recorded. In addition, participants could stop participating in the study and abandon the questionnaire at any time before the end of the questions.

### Statistical analysis

Data distribution was assessed using Shapiro–Wilk’s test. Continuous variables were reported as mean and standard deviation (SD) or by median and interquartile range (IQR) within squared brackets according to their distribution. The comparison between continuous variables was carried out through Mann–Whitney, Kruskal Wallis tests, or Student’s *T* test, where applicable. Categorical variables were presented as numbers, and relative percentages and their distribution among groups were assessed through chi-squared and Fisher’s exact tests. To explore the comorbidities representing possible predictors of MG rather than HC, an unconditional logistic regression analysis was performed for each study variable. The odds ratios (OR) with 95% confidence intervals (CI) and *p* value (two-tailed test, *α* = 0.05) were calculated. Multivariate analysis was performed to investigate the independent effect of a risk or protective factor after adjustment for one or several other factors or to adjust for confounding variables. Parameters associated with the outcome at the univariate analysis with a threshold of *p* = 0.10 were included in the multivariate model. Age and sex were considered an a priori confounders, regardless of the level of significance. The model was manually constructed using the likelihood ratio test (LRT) to compare the log-likelihood of the model with and without a specific variable. Likewise, other associations between putative independent predictors and the dependent variable were assessed by performing univariate and multivariate logistic regression models. All statistical analyses were performed by using SPSS (IBM Corp. Released 2019. IBM SPSS Statistics for MacOS, Version 26.0. Armonk, NY: IBM Corp). For all the statistical tests, the level of significance has been set at *p* < 0.05.

## Results

One hundred ninety-four MG patients have been evaluated for study inclusion. Sixteen MG patients have been excluded due to incomplete medical records; hence, 178 patients affected by MG (46% female, mean age 59.2 ± 15) were enrolled while 441 HC (55% female, median age 49 years [30–60] completed the web-based questionnaire; after matching procedures according to the matching criteria, a total of 178 HCs were included in the final analysis (Table 1). All continuous variables showed a not-normal distribution according to the Shapiro–Wilk’s test (all *p* < 0.05) except age (*p* = 0.13) and number of comorbidities (*p* = 0.3).

**Table 1:** Demographic features of the study population and comparison of the comorbidity’s frequency between and subjects with myasthenia gravis and healthy controls

	Myasthenia gravis *N* = 178	Healthy controls *N* = 178	*p*
General features			
Females, *n* (%)	81 (45.5)	81 (45.5)	> 0.05
Age, mean ± SD, years	59.2 ± 15	58.5 ± 12.5	0.7
Presence of comorbidity, *n* (%)	153 (86)	135 (75.8)	0.02
Two or more comorbidities, *n* (%)	119 (65.7)	87 (48.9)	0.001
No. of comorbidities, mean ± SD	2.5 ± 1.9	1.96 ± 1.9	0.004
Non-autoimmune comorbidity, *n* (%)			
Hypertension	64 (35.9)	58 (32.6)	0.5
Diabetes	28 (15.7)	11 (6.2)	0.004
Osteoporosis	34 (19.1)	24 (13.5)	0.2
Hypercholesterolemia	23 (12.9)	45 (25.3)	0.003
Thyroid disease	13 (7.3)	17 (9.6)	0.4
Kidney disease	13 (7.3)	12 (6.7)	0.8
Cardiovascular	41 (23.0)	21 (11.8)	0.005
Ocular disease	14 (7.9)	23 (12.9)	0.1
Gastrointestinal	24 (13.5)	41 (23)	0.02
Hematological	17 (9.6)	8 (4.5)	0.06
Non-thymic tumors	28 (15.7)	16 (9.0)	0.05
Respiratory	23 (12.9)	6 (3.4)	0.001
Neurological	59 (33.1)	18 (10.1)	< 0.0001
Psychiatric	27 (15.2%)	27 (15.2%)	1
Autoimmune, *n* (%)	44 (24.7)	21 (11.8)	0.002

Abbreviations: *SD*, standard deviation

### Frequency of comorbidities in myasthenia gravis subjects and healthy controls

After the matching procedures, female sex (45.5% vs 45.5%) and age (59.2 ± 15 years vs 58.5 ± 12.5) did not differ between MG patients and HCs (all *p* > 0.05). MG patients showed a higher frequency of comorbidity (86% vs 75.8%, *p* = 0.02) and multimorbidity (65.7% vs 48.9%, *p* = 0.001) as well as a higher number of comorbidities per person 2.5 ± 1.9 vs 1.96 ± 1.9, *p* = 0.004). The frequency of comorbidity in MG patients and HCs is reported in Table 1. At univariate analysis, diabetes mellitus, cardiovascular, respiratory, neurological, and autoimmune diseases were associated with MG (all *p* < 0.05) while, after multivariate analysis, only cardiovascular, respiratory, neurological, and autoimmune diseases were still significantly associated with MG (all *p* < 0.05; Table 2). On the contrary, results from the multivariate analysis showed that hypercholesterolaemia, ocular diseases, and gastrointestinal diseases were significantly associated with HCs (all *p* < 0.05) (Table 2). Considering the single comorbidity, GERD was more frequent in HC than MG (*p* = 0.001), while gallstones (*p* = 0.03), COPD (*p* = 0.006), stroke/TIA (*p* = 0.01), epilepsy (*p* = 0.03), neuropathy (*p* = 0.005), and Hashimoto’s thyroiditis (*p* = 0.04) were more common in MG than in HC (Supplementary file 2).

**Table 2:** Univariate and Multivariate analyses exploring the comorbidity associated with myasthenia gravis by adjusting for sex and age

	Univariate analysis			Multivariate analysis		
	OR	95% CI	*p* value	AdjORs	95% CI	*p* value
Demographic features						
Male vs female (ref)	1	0.7–1.5	1	0.9	0.5–1.5	0.7
Age, y	1	0.99–1.02	0.7	1	0.99–1.02	0.9
Comorbidities						
Hypertension	1.2	0.75–1.8	0.5			
Diabetes	2.8	1.4–5.9	0.005	2.3	0.9–5.5	0.05
Osteoporosis	1.5	0.86–2.68	0.15			
Hypercholesterolemia	0.4	0.25–0.76	0.003	0.4	0.2–0.7	0.002
Thyroid	0.8	0.4–1.6	0.4			
Kidney	1.2	0.53–2.63	0.68			
Cardiovascular	2.2	1.3–3.9	0.006	2.6	1.3–5.3	0.009
Ocular	0.6	0.29–1.16	0.1	0.4	0.17–0.9	0.03
Gastrointestinal	0.5	0.3–0.9	0.02	0.3	0.2–0.9	0.001
Hematologic	2.2	0.9–5.3	0.07	2.1	0.8–5.7	0.1
Non-thymic tumors	1.9	0.98–3.6	0.06	1.4	0.7–2.9	0.4
Respiratory	4.3	1.7–10.7	0.002	4	1.4–11.1	0.009
Neurological	4.4	2.5–7.9	< 0.0001	5.3	2.7–10.3	< 0.0001
Psychiatric	1	0.56–1.8	1			
Autoimmune	2.5	1.4–4.3	0.002	2.5	1.2–4.9	0.01

Abbreviations: *OR*, odds ratios; *CI*, confidence intervals; *AdjORs*, adjusted odds ratios

### Distribution of comorbidities according to age at MG onset

**Fig. 1:**
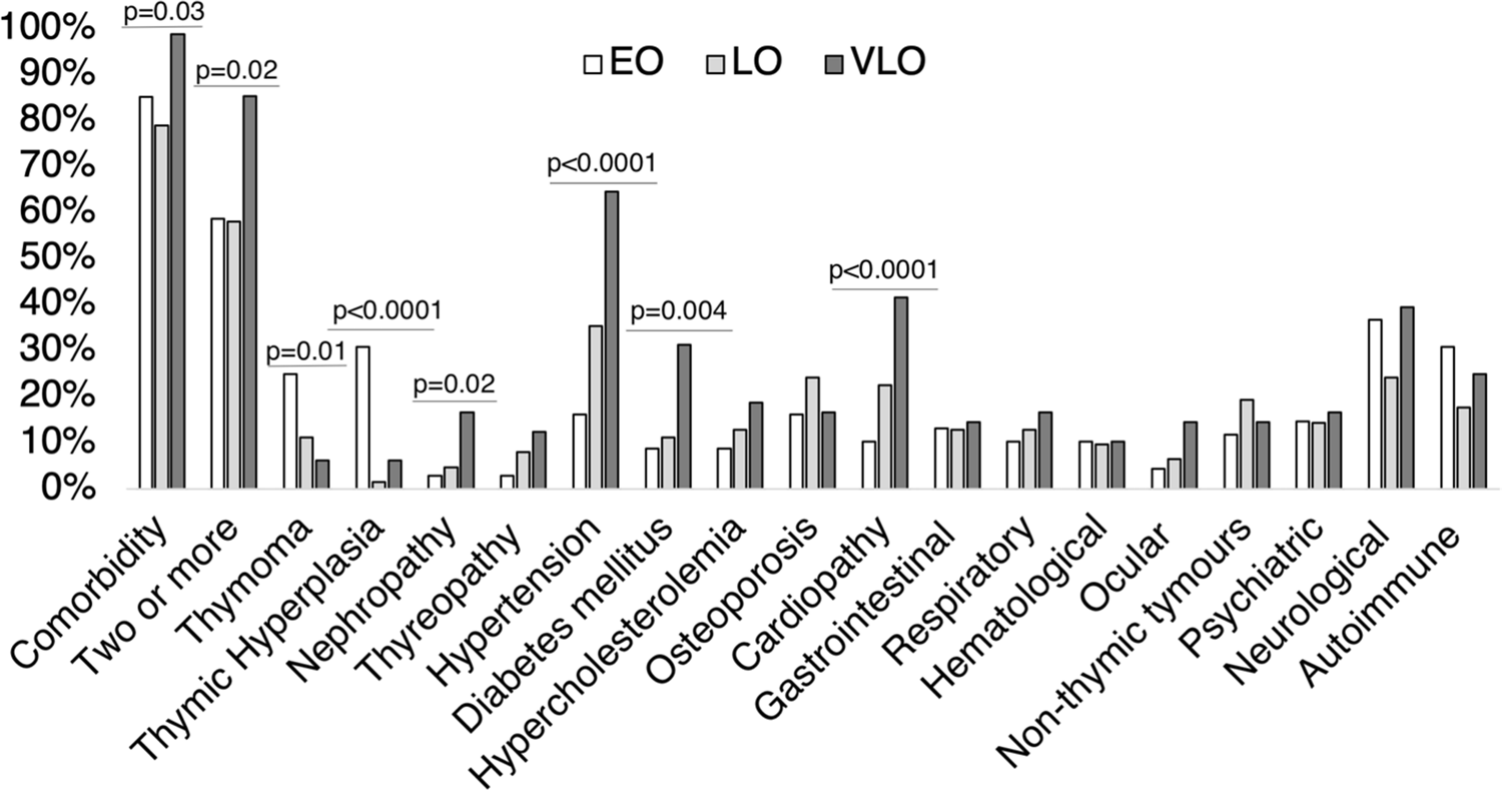
Distribution of comorbidities in MG patients according to age at disease onset. Abbreviations: EO, early onset; LO, late onset; VLO, very late onset

VLOMG patients had higher frequency of comorbidity (95.8%, *p* = 0.03) and multimorbidity (85.4%, *p* = 0.02) compared to LOMG (79% and 58.1%) and EOMG (85.3% and 58.8%). VLOMG showed also a higher frequency of hypertension (64.6% vs 35.5% vs 16.20%; *p* < 0.0001), diabetes mellitus (31.3% vs 11.3% vs 8.8%, *p* = 0.04), cardiopathies (41.7% vs 22.6% vs 10.3%; *p* < 0.0001), and nephropathies (16.7% vs 4.8% vs 2.9%; *p* = 0.02) compared, respectively, to patients with LOMG and EOMG (Fig. 1). Conversely, thymoma and thymic hyperplasia were significantly more common in patients with EOMG (25% and 39.9%, respectively) compared to those with LOMG (11.3% and 1.6%, respectively) and VLOMG (6.3% and 6.3%, respectively), and this was statistically significant for both thymoma (*p* = 0.01) and thymic hyperplasia (*p* < 0.0001) (Fig. 1). There were no other statistically significant differences between groups in the frequency of comorbidities according to the age of MG onset. Subsequently, logistic regression was conducted to assess the specific comorbidities that were associated with MG according to age at disease onset (due to a small sample of some comorbidity groups, these analyses were conducted by stratifying subjects into EOMG vs LOMG, and the latter was considered the reference category) (Table 3). At univariate analysis, only thymoma and thymic hyperplasia were found to be associated with EOMG (all *p* < 0.05), whereas hypertension and cardiovascular diseases were associated with LOMG (all *p* < 0.05). After multivariate analysis, thymoma and thymic hyperplasia remained strongly and significantly associated with EOMG (all *p* < 0.05; Table 3).

**Table 3:** Univariate and multivariate analyses exploring the comorbidity predictors of early-onset myasthenia gravis (MG) (reference category late-onset MG) by adjusting for sex and type of MG

	Univariate analysis			Multivariate analysis		
	OR	95% CI	*p* value	AdjORs	95% CI	*p* value
General features						
Male vs female (ref)	0.28	0.1–0.5	< 0.0001	0.5	0.2–1	0.07
Generalized vs ocular MG (ref)	0.9	0.5–1.7	0.8	0.9	0.4–2.1	0.8
Comorbidities						
Thymoma	3.3	1.4–7.8	0.006	4.1	1.5–11.1	0.006
Thymic hyperplasia	11.8	3.8–36.4	< 0.0001	11.1	3.1–39.8	< 0.0001
Hypertension	0.2	0.1–0.4	< 0.0001	0.4	0.2–1.1	0.07
Diabetes	0.4	0.15–1	0.05	0.7	0.2–2.4	0.6
Osteoporosis	0.7	0.3–1.6	0.4			
Hypercholesterolemia	0.5	0.2–1.4	0.2			
Thyroid	0.3	0.06–1.36	0.1	0.3	0.05–1.4	0.1
Kidney	0.3	0.06–1.3	0.1	0.5	0.1–3	0.4
Cardiovascular	0.3	0.1–0.6	0.002	0.4	0.1–1.1	0.09
Ocular	0.4	0.1–1.5	0.2			
Gastrointestinal	0.9	0.4–2.3	0.9			
Hematologic	1.1	0.4–3.2	0.8			
Non-thymic tumors	0.6	0.2–1.5	0.3			
Respiratory	0.7	0.3–1.7	0.4			
Neurological	1.3	0.7–2.5	0.4			
Psychiatric	0.9	0.4–2.2	0.9			
Autoimmune	1.7	0.8–3.4	0.1	2	0.8–5	0.1

Abbreviations: *OR*, odds ratios; *CI*, confidence intervals; *AdjORs*, adjusted odds ratios

### Distribution of comorbidities according to sex of MG patients

**Fig. 2:**
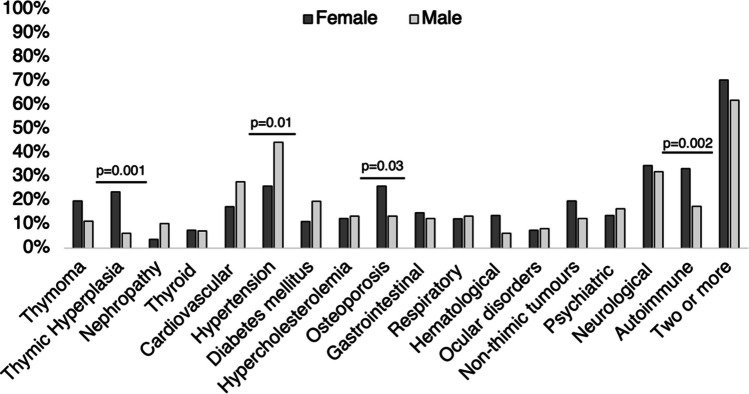
Distribution of the comorbidities according to sex in patients with myasthenia gravis

The frequency of comorbidities did not differ between males and females (86.4% and 85.6%; *p* = 0.9), although males were more affected by hypertension (44.3% vs 25.9%; *p* = 0.01), while females showed a higher prevalence of thymic hyperplasia (23.5% vs 6.2%; *p* = 0.001), autoimmune diseases (33.3% vs 17.5%; *p* = 0.02), and osteoporosis (25.9% vs 13.4%; *p* = 0.03). Cardiovascular disease showed a slightly higher frequency in males than females (27.8% vs 17.3; *p* = 0.09), although only ischemic cardiopathy reached the statistical significance among this category (10.3% vs 2.5%; *p* = 0.04). Neurological diseases were similarly distributed in males and females (Fig. 2), but migraine showed a significantly higher prevalence in females (14.8% vs 2.1%; *p* = 0.002). No other statistically significant differences were reported. The complete comparisons of the comorbidity frequency between MG male and female are reported in Fig. 2.

### Comorbidities associated with generalized myasthenia gravis

**Fig. 3:**
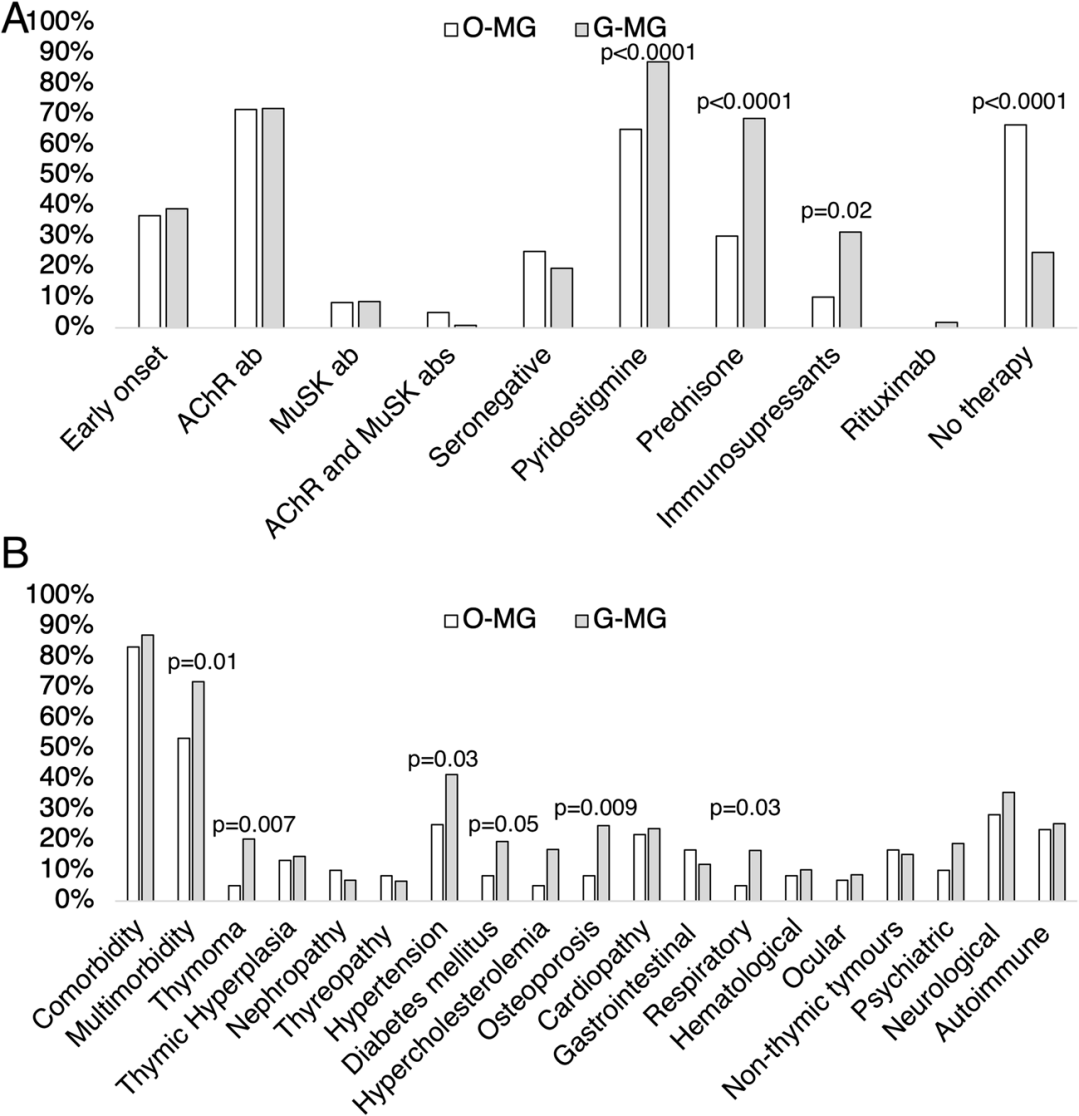
Comparison of the clinical features (**A**) and of comorbidities frequency (**B**) between ocular MG and generalized MG

Sixty (33.7%) out of 178 subjects with MG were classified as OMG (MGFA Class I), while the remaining 118 (72.7%) were GMG (MGFA Class II, III, IV, or V). Out of 178 MG subjects, *n* = 127 (71.3%) were positive for anti-AChR antibodies (median serum titer 6.4 nmol/l [1.4–19.9]) and 15 (8.4) for anti-MuSK antibodies (median serum titer 1 U/ml [0.76–1.97]). Thirty-nine (21.3%) MG subjects were double-seronegative. Patients with OMG and GMG did not differ for age at the time of the study (median 58.5 years [50–68] vs 60.5 years [51–72]; *p* = 0.2) although a slightly higher proportion of females was found in GMG (50.8% vs 35%; *p* = 0.05) as well as patients with GMG had a higher disease duration (40.7 months [14–95] vs 25.7 [6–46]; *p* = 0.006). The relevant difference between patients with OMG and GMG concerned the therapies received by patients and are represented in Fig. 3A. The frequency of comorbidity was similar between GMG and OMG (87.3% vs 83.3%; *p* = 0.5) although the frequency of multimorbidity was higher in GMG (72% vs 53.3%; *p* = 0.01). Thymoma, diabetes mellitus, and psychiatric disease were more common in GMG without other differences between groups (all *p* < 0.05; Fig. 3B). At univariate analysis, thymoma, hypertension, osteoporosis, hypercholesterolemia, and respiratory diseases were significantly associated with the GMG subtype compared with OMG (all *p* < 0.05; Table 4), while after multivariate analysis only the presence of thymoma remained significantly associated with GMG subtype while male sex was associated with OMG (*p* = 0.03; Table 4).

**Table 4:** Univariate and multivariate analyses exploring the comorbidity associated with generalized myasthenia gravis (reference category ocular myasthenia gravis) by adjusting for sex, age, and disease duration

	Univariate analysis			Multivariate analysis		
	OR	95% CI	*p* value	AdjORs	95% CI	*p* value
Demographic features						
Male vs Female (ref)	0.52	0.3–0.99	0.46	0.4	0.2–0.9	0.03
Age, years	1	0.99–1.03	0.2	1	0.98–1.04	0.3
Disease duration, months	0.9	0.5–1.7	0.8	1	0.99–1	0.4
Comorbidities						
Thymoma	4.9	1.4–16.9	0.01	5.8	1.5–22	0.009
Thymic hyperplasia	1.1	0.4–2.7	0.8			
Hypertension	2.1	1.1–4.2	0.03	1.8	0.7–4.2	0.2
Diabetes	2.7	0.96–7.4	0.06	2.4	0.8–7.3	0.1
Osteoporosis	3.6	1.3–9.8	0.01	1.8	0.6–5.4	0.3
Hypercholesterolemia	3.9	1.1–13.6	0.04	2.3	0.6–9.1	0.2
Thyroid diseases	0.8	0.3–2.6	0.71			
Kidney diseases	0.57	0.2–1.8	0.3			
Cardiovascular diseases	1.1	0.5–2.4	0.8			
Ocular diseases	1.3	0.4–4.3	0.7			
Gastrointestinal diseases	0.7	0.3–1.6	0.4			
Hematologic diseases	1.2	0.4–3.7	0.7			
Non-thymic tumors	0.9	0.4–2	0.8			
Respiratory diseases	3.9	1.1–13.6	0.04	3	0.8–11.5	0.1
Neurological diseases	1.4	0.7–2.7	0.3			
Psychiatric diseases	1.9	0.7–5.1	0.2			
Autoimmune diseases	1.1	0.5–2.3	0.8			

Abbreviation: *OR*, odds ratios; *CI*, confidence intervals; *AdjORs*, adjusted odds ratios; *y*, years; *m*, months

### Comorbidities and MGFA class

**Fig. 4:**
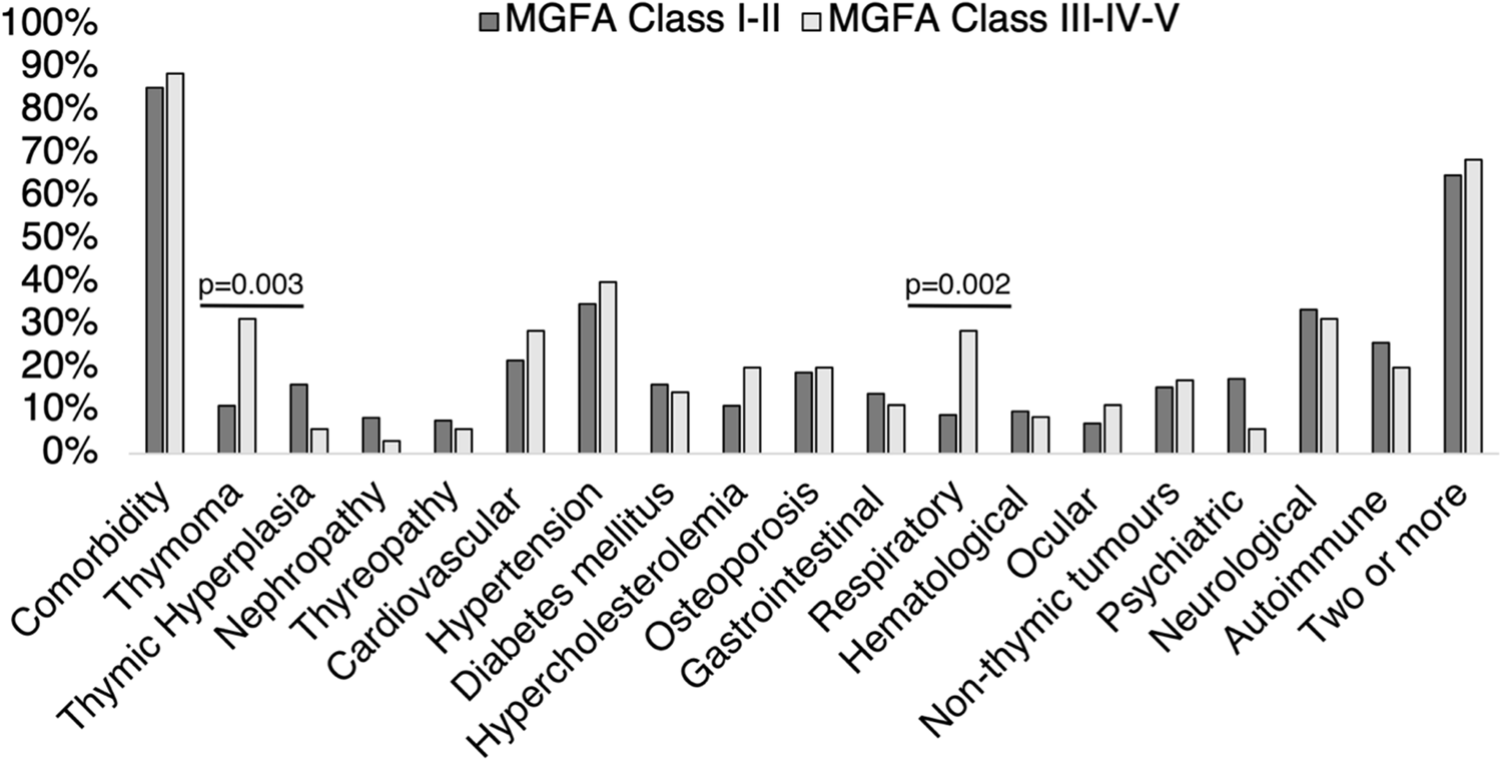
Distribution of the comorbidities between MGFA Class I–II and III–IV–V. Abbreviations: MGFA, Myasthenia Gravis Foundation America

Out of 178 MG subjects,143 (80.4%) were classified as having a grade MGFA Class I or II, while the remaining 35 (19.6%) had a grade MGFA III, IV, or V. Overall, there was no significant difference in the prevalence of comorbidities between these two groups (85.3% vs 88.6%; *p* = 0.6) although subjects in the MGFA Class III, IV, and V showed a higher frequency of respiratory diseases (28.6% vs 9.1%; *p* = 0.002) and thymoma (31.4% vs 11.2%; *p* = 0.003). The complete distribution of comorbidities across MGFA classes is reported in Fig. 4. After multivariate logistic regression analysis, respiratory diseases (*p* = 0.002; OR 4.9 CI 95%: 1.8–13.9) and thymoma (*p* = 0.04; OR 4.3 CI 95%: 1.6–11.3) were significantly associated with higher MGFA Class. Exploring specific comorbidities under the category of respiratory diseases, patients with MGFA Class III–IV and V showed a higher frequency of OSAS (2.1% vs 2.4%; *p* = 0.03) and asthma (2.8% vs 11.4%; *p* = 0.049) compared to patients with MGFA Class I or II.

### Comorbidities and medication

Overall, the most used drug in our cohort was pyridostigmine (80%), followed by prednisone (56%). GMG subjects showed a higher disease duration (*p* = 0.006) than OMG, and a higher percentage of them were treated with pyridostigmine (*p* < 0.0001), prednisone (*p* < 0.0001), and immunosuppressants (*p* < 0.0001) (Fig. 3A). Pyridostigmine and steroids were similarly prescribed in OMG and GMG, while azathioprine and immunosuppressants were more frequently taken by GMG patients (Fig. 3A). Osteoporosis (*p* = 0.001), neuropathy (*p* = 0.08), and psychiatric diseases (*p* = 0.08) were more common in patients taking azathioprine and prednisone. Respiratory diseases were less common in patients taking no medication (*p* = 0.007) and more common in those taking prednisone (*p* = 0.019).

## Discussion

This study evaluated the association of comorbidities in a relatively large cohort of MG patients compared to age- and sex-matched healthy controls from two different specialized neuromuscular clinics. Comorbidities were present in 87% of MG patients, similarly to previous studies [11].

### Comorbidities in MG vs HC

Overall, hypertension, neurological disease, and autoimmune disease were found as the most prevalent comorbidities (from 26 to 36%) in MG patients; furthermore, thyroid disorders, heart disease, osteoporosis, gastrointestinal, psychiatric disorders, and diabetes were also quite frequent, affecting approximately 15–20% of patients. Overall, these data confirm that MG patients are more prone to autoimmune comorbidities than nonmyasthenic subjects (*p* < 0.0001), especially in females [18] (Fig. 2). Hence, autoimmune and non-autoimmune comorbidities deserve specific considerations [8, 10, 16, 19, 23–25].

#### Autoimmune comorbidities

Several studies reported that about 13% of MG patients develop other autoimmune diseases (AD) [17, 23]. MG has been linked to several autoimmune disorders, including autoimmune thyroid disorders, systemic lupus erythematosus, and rheumatoid arthritis[25]. It was recently reported that 10% of MG had autoimmune comorbidities and 21% showed autoantibodies (anti-TPO, ANA, anti-dsDNA, rheumatoid factor) in the absence of clinical evidence of AD [11]; therefore, the prevalence of this comorbidity may depend on the duration of follow-up. In a Chinese study [17], AD was more frequent in patients without thymoma and with mild disease severity. In our cohort, AD was reported in 25% of MG patients, showing a significant association with MG, and being more frequent in females than males; these data confirm earlier data described by Ozdemir et al. [8], who reported AD as the third most common type of comorbidity with a frequency of 25%; similarly, data from a large Swedish population–based study [26] showed that 22% of MG had at least one diagnosis of AD, especially among young women.

Regarding disease severity assessed by MGFA, we cannot confirm the differences reported by the Chinese study [17] in the frequency of AD among OMG and GMG, in the different degrees of MGFA (Fig. 4), nor in the age of disease onset (Fig. 1) or antibody status. However, these differences might be explained by the limited number of OMG patients in our study and different genetic susceptibility to autoimmune disorders in Eastern countries. Concerning thyroid disorders, described in 10–18% of MG patients from previous studies [23, 25], were reported in 22% of patients in the present research and were more frequent in females and in ocular MG. Moreover, autoimmune thyroid disorders were recognized in a subgroup of 15% of patients. Some common features, triggers, genetic susceptibility, and pathogenesis that characterize autoimmune thyroid disease and MG could explain the overlap between these disorders [27]. Among thyroid disorders, autoimmune thyroiditis was the most frequent subtype, confirming data from previous studies [17, 26], in which the overall frequency of hypothyroidism among MG patients and the control group of the population without MG was four times higher. Interestingly, a strong association between MG and polymyositis/dermatomyositis, systemic lupus erythematosus, and Addison’s disease has been described, suggesting a potentially relevant connection with HLA B8-DR3-related diseases [26].

#### Non-autoimmune comorbidities

Hypertension was the most frequent comorbidity in this cohort, as in previous reports [8, 10, 11, 16], although there was no significant difference compared with controls. Surprisingly, neurological comorbidity was found to be the second most frequently comorbidity described (34%) and the one associated with the highest risk of MG. There are very few reports exploring the presence of neurological comorbidity in MG, although recent data on this topic has been described [8, 28]. This high prevalence of neurological comorbidity in MG is a new finding, as previous reports had described only restless leg syndrome (RLS), stroke, and migraine in 5%, 6%, and 6% of cases, respectively, which collectively accounted for 17% of MG patients [11]. In the study by Ozdemir et al., the authors describe neurological disease in about 4% of patients describing rare cases of stroke, carpal tunnel syndrome, epilepsy, and CIDP. The risk of ascertainment bias cannot be excluded from neurologic comorbidity, as MG patients consult a neurologist more regularly than HC. According to our findings, neuropathy was the most common comorbidity among neurological comorbidities. Peripheral neuropathy may follow diabetes, nutritional deficiencies, autoimmune diseases, and hypothyroidism, most of which often coexist with myasthenia gravis; therefore, peripheral neuropathy is likely to complicate the clinical course of MG. However, there are cases of association between MG and CIDP [8]. Moreover, many symptoms of advanced disease in MG, such as weakness, pain, and muscle wasting, may resemble myelopathy, radiculopathy, or myositis [29, 30]. Therefore, it is crucial to draw attention to confusing clinical presentations such as stable limb weakness, ocular symptoms, dysphagia, and dysarthria, which can be observed in both MG and neuromuscular disorders. Another interesting finding is the increased incidence of stroke, which might be related to arrhythmia (i.e., atrial fibrillation) and cardiopathy. Interestingly, epilepsy has not yet been studied extensively in MG patients, although our prevalence (3%) was much higher than that reported in a recent Norwegian study [31]; this finding emphasizes the need for a deeper understanding of the pathophysiological mechanisms presumably underlying both epilepsies and autoimmune diseases. Thus, further studies exploring neurological comorbidity in MG patients are needed. Finally, although psychiatric comorbidity has been widely reported in MG [28, 32–35], no significant differences with HC were found in the present study.

Regarding diabetes, the prevalence of diabetes was significantly higher in VLOMG than in LO and EO disease, suggesting a more likely correlation with type II diabetes rather than autoimmune diabetes. An even higher frequency of diabetes has been reported by Ozdemir et al. [8], being the second most common comorbidity in that study; the authors suggested that the excessive use of corticosteroids might interfere with glucose control, thus causing an increased risk of diabetes [8]. In any case, the relationship between diabetes and MG is controversial: in addition to in vivo studies suggesting that diabetes can promote both adaptive and innate immunity and exacerbate the clinical symptoms of MG [36, 37], there is evidence that chronic use of steroids to treat MG adversely affects glico-metabolic balance [38, 39].

Cardiac involvement in MG has already been described, also supported by increased use of drugs active in cardiovascular symptoms [40, 41], but the cause of heart disease in MG is currently unknown. Heart diseases, including coronary artery disease and atrial fibrillation and arrhythmias, have been recently reported in MG [8]. It is noteworthy that antibodies against cardiac muscle have been reported in MG sera [42], while hypertension could partially explain this comorbidity [11, 43]. However, in the present study, the frequency of hypertension did not differ in MG compared to HC, while ventricular hypertrophy was more frequent in MG, although this finding was not significant. It has been shown that baroreflex sensitivity can be reduced in MG patients through various mechanisms; such dysfunction can lead to dizziness, syncope, atrioventricular block, ventricular and supraventricular arrhythmias, QT prolongation, and even atrial fibrillation [44]. Data from the present study confirm the presence of arrhythmias in 6.5% of MG [37], although the latter does not appear to have a higher frequency in MG patients than in HC. Concerning lipid alterations, we found that hypercholesterolemia was more common in HC in contrast with a previous report [10]. It should be kept in mind that beta-blockers can unmask MG [45, 46]: from this point of view, there is a possibility that patients with cardiovascular risk factors (i.e., hypertension, hypercholesterolemia, etc.) are more likely to arrive at the diagnosis of MG than other patients not taking this kind of medication. These considerations could partially explain the increased rate of heart disease in MG found in the present study, although further studies are needed to confirm this data.

Among respiratory diseases, COPD was the most common comorbidity (6%), followed by asthma (5%) reported in the present study in patients with MG. COPD was reported by Ozdemir in 2% of patients [8]; previous studies (mainly case reports) have described the association between MG and severe non-atopic asthma [47], underscoring the need to overlook neuromuscular disorders, such as MG, in patients experiencing acute asthma, especially when there is difficulty in tapering steroids [48]. Moreover, the association between MGFA class III–V and respiratory diseases should be emphasized especially in the case of treatable causes; this is the case with OSAS and asthma, which should be observed carefully, due to the risk of misdiagnosis with MG exacerbation in GMG. In these complicated cases, respiratory failure could follow exacerbations in patients already compromised by chronic respiratory failure, with fatal outcome [7].

Finally, MG patients in the present research reported an overall increased frequency of malignancy than HC, although this finding was not statistically significant with similar frequency reported by Vijayan and Ozdemir [8, 10]; the risk of immunosuppression from azathioprine, steroids, and B-depleting therapies could explain the increased prevalence of neoplasms in MG [13]. However, some recent evidence linked some genetic predisposing to carcinogenesis in some MG patients [49], suggesting the need for careful prevention and screening for cancer in these fragile patients.

### Comorbidities and age at disease onset

Patients with VLOMG reported the highest frequency of comorbidities (97%), which is much higher than how reported previously in this subtype [10]. Indeed, VLOMG patients presented with several age-related comorbidities (hypertension, diabetes, cardiopathy, ocular and kidney disease) in addition to the typical MG-specific comorbidities (Fig. 1). These confirmatory data enhance the role of comorbidity in older patients [8, 16, 19, 24], especially cardiovascular risk factors (hypertension, diabetes), that might lead to the administration of medication that may worsen MG such as beta-blockers or statins, as well as a lesser appreciation of myasthenic symptoms [10, 16]. It should be noted that, in the present study, both arrhythmias and ischemic cardiomyopathy were more prevalent in patients with VLOMG than in those with LOMG and EOMG (40% vs 20% and 9% respectively), this finding is a novelty, and it might have been underestimated in previous studies [8, 10, 16]. Moreover, the role of aging in the pathophysiology of these comorbidities is supported by the fact that they accumulate with increasing patient age from EOMG to VLOMG (Fig. 1) [10, 16]. In contrast, EOMG patients had thymoma and thymic hyperplasia more often, with a low rate of cardiovascular comorbidity. Some authors suggested that EOMG seems to have a higher probability of having thyroid diseases than LOMG [25], but our data do not confirm this finding (Fig. 1). Of interest, and as previously reported [16], we did not find a higher prevalence of autoimmune disorders in EOMG than in LOMG or VLOMG. Therefore, we hypothesize that most comorbidities are associated with age rather than age at disease onset, with the only exception of thymoma and thymic hyperplasia which are almost exclusive to EOMG.

### Comorbidities and sex

In line with previous studies, autoimmune comorbidities were more common in females (Fig. 2). Also, females were more prone to osteoporosis and thymic hyperplasia but had a lower incidence of hypertension than males [8, 10, 11, 16, 23].

### Comorbidities and MG subtype (OMG vs GMG) and MGFA

Concerning the severity of MG, patients with GMG often had two or more comorbidities in about three-quarters of cases. This could be related to a greater need for immunosuppressant drugs, steroids, and combination therapies in GMG patients (Fig. 3). The latter patients had higher rates of thymoma, respiratory disease, hypercholesterolemia, osteoporosis, and hypertension than OMG. Furthermore, thymoma and respiratory disorders were more prevalent in MGFA class III-IV and V.

### Comorbidities and therapy

Osteoporosis, neuropathy, and psychiatric diseases were associated with the use of azathioprine and steroids, while ocular comorbidity was associated with the use of steroids. However, a correlation between the dosage of steroids and the presence of diabetes, hypertension, and osteoporosis was not confirmed in this cohort. However, the relationship between comorbidity and therapy is difficult to explore for several reasons: first, because of selection bias in elderly patients, since it is well-known that the number of medications increases with aging; second, considering the specific attention paid in the choice of therapy in the presence of comorbidity. Therefore, we do not know whether comorbidity was antecedent or after medication use. Prospective studies are needed to clarify this point. Finally, although the steroid dosage used for the analysis was the last one used (maintenance), this may not reflect the overall steroid exposure since diagnosis in MG patients with a long duration of disease. Conversely, respiratory comorbidity needs some special considerations. Indeed, untreated or undertreated MG patients had higher rates of respiratory comorbidity. This fact underlines the need for adequate immunosuppressive treatment in MG to reduce the risk of respiratory impairment.

### Strengths and limitations

The major strength of this study lies in the comparison of comorbidities grouped according to specific disease groups in a relatively large multicentric, outpatient cohort of patients affected by MG and healthy controls selected from the general population; in fact, most of the studies conducted to date are retrospective and include only patients with MG without a control group [10, 25, 40, 50]. For this purpose, we conducted a case–control analysis by matching each MG patient with a respective age- and sex-matched control. This analysis made it possible the identification of comorbidities specific for MG, which therefore require special attention in disease management. Another strength of this study is the categorization of patients according to age at disease onset, focusing not only on EOMG and LOMG, as in previous studies [8, 16], but with a specific focus on VLOMG, as emerged from the recent debate [19, 24]. In addition, a stratification by disease severity based on MGFA was calculated in multivariate analysis, as frequently suggested by expert colleagues [24]. Nonetheless, some limitations should be considered when interpreting our data. First, while comorbidities in patients with MG were collected using data from clinical records, data from HC were collected via a web-based questionnaire, and therefore, a possible recall bias cannot be excluded. Furthermore, certain diseases diagnosed by blood parameters (e.g., borderline diabetes, dyslipidaemia, anemia) may have been underestimated in the HC group. Similarly, psychiatric and infectious comorbidity might be underrated in both MG and HC subjects due to the lack of systematic evaluation (e.g., neuropsychological evaluations, assessment of psychiatric symptoms, serological tests). Second, our clinic is a predominantly outpatient tertiary referral clinic; therefore, more severe cases with admission to hospitals other than ours and intensive care unit admission may have been not included in this cohort. Third, this study did not focus on the association between myasthenic crisis and comorbidity. Furthermore, although association analyses were controlled for demographics, residual confounding (e.g., medical, and neuropsychiatric comorbidity, use of specific drugs) cannot be excluded. Finally, due to the cross-sectional study design, a relationship between MG and specific types of comorbidities can only be hypothesized. Accordingly, further perspective studies on large cohorts are needed to describe the actual impact of comorbidity in MG.

## Conclusions

Today, patients with MG have a good prognosis and life expectancy close to that of the general population; however, the management of fluctuating symptoms and refractory cases remains challenging, and comorbidities can significantly influence the choice of appropriate treatments. Physicians should be able to control myasthenic symptoms while preventing the adverse effects of long-term immunosuppressive therapies. Furthermore, early detection of comorbidities could allow specific treatments and preventive measures to improve the overall care of MG patients. Overall, although preliminary, data of the present study underline the complexity of MG and the need for a multidisciplinary approach in the management of the disease, involving not only neurologists, but also other specialists (i.e., cardiologist, pneumologists and hematologist) within the framework of personalized medicine. A longitudinal follow-up of this cohort, as well as other large prospective studies, will help to better understand the prognostic role of systemic comorbidities in MG.

### Supplementary Information

Below is the link to the electronic supplementary material.

Supplementary file1 (DOCX 23 KB)

Supplementary file2 (DOCX 28 KB)

## Data Availability

Data are available from the corresponding author upon reasonable request.
