# Supplementary material for: Comorbidity in myasthenia gravis: multicentric, hospital-based, and controlled study of 178 Italian patients
Source: Neurol Sci. 2024 Feb 22;45(7):3481–94. doi: 10.1007/s10072-024-07368-0 (PMC11176220; doi:10.1007/s10072-024-07368-0)
Supplement: Supplementary file 1 — Supplementary file1 (DOCX 23 KB) [file 10072_2024_7368_MOESM1_ESM.docx]

**- Supplementary File 1 -**

**Retrospective observational study on disease frequency in the general population and in patients with Myasthenia Gravis.**

*The following questionnaire is collected anonymously, therefore you should not write your name and/or surname at any point.*

***Legend:*** ******* *Mandatory field*

1. ******* The aim of this questionnaire is to collect information on the presence or absence of pathologies (such as thyroid disorders, nephrological disorders, hypertension, diabetes, osteoporosis, autoimmune disorders, hypercholesterolaemia, cardiovascular, gastrointestinal, respiratory, oncological, haematological, neurological and psychiatric disorders) in subjects aged 18 years or over and not affected by myasthenia gravis. The subjects recruited by means of this questionnaire will constitute a healthy control population in the retrospective observational study aimed at comparing the frequency of the aforementioned pathologies between myasthenia gravis patients and unaffected subjects.

Do you agree to participate in the study? *The answer "I do not give consent" will terminate automatically end the questionnaire and data collection.*

- I give consent
- I do not give consent

1. ******* Sex (male/female)?

- Male
- Female

1. ******* Please write your age below:

______________

1. ******* Do you suffer from kidney disorders (kidney stones, glomerulonephritis, kidney disease, etc.)?
   - Yes
   - No
2. ******* If you answered “yes” to the previous question, please state below which ones:

___________________________________________________________

___________________________________________________________

1. ******* Do you suffer from autoimmune diseases (*e.g.*, systemic lupus erythematosus, vasculitis, myasthenia gravis, multiple sclerosis, rheumatoid arthritis, Hashimoto's thyroiditis, etc.)?
   - Yes
   - No
2. ******* If you answered “yes” to the previous question, please state below which ones:

___________________________________________________________

___________________________________________________________

1. ******* Do you suffer from thyroid diseases (*e.g.*, nodules, hypothyroidism hyperthyroidism, etc.)?
   - Yes
   - No
2. ******* If you answered “yes” to the previous question, please state below which ones:

___________________________________________________________

___________________________________________________________

1. ******* Do you suffer from high blood pressure?
   - Yes
   - No
2. ******* Do you suffer from hypercholesterolaemia?
   - Yes
   - No
3. ******* Do you suffer from cardiovascular diseases (*e.g.*, previous heart attack, heart disease, arrhythmias, etc.)?
   - Yes
   - No
4. ******* If you answered “yes” to the previous question, please state below which ones:

___________________________________________________________

_____________________________________________________________________

1. ******* Do you suffer from osteoporosis? *
   - Yes
   - No
2. ******* Do you suffer from eye diseases (e.g., cataracts, conjunctivitis, glaucoma, etc.)? (Excluding astigmatism, myopia, hypermetropia)
   - Yes
   - No
3. ******* If you answered “yes” to the previous question, please state below which ones:

___________________________________________________________

_____________________________________________________________________

1. ******* Do you suffer from a gastrointestinal disease (*e.g.*, gastroesophageal reflux, gastritis, Chron's disease, ulcerative rectocolitis, etc.)?
   - Yes
   - No
2. ******* If you answered “yes” to the previous question, please state below which ones:

___________________________________________________________

_____________________________________________________________________

1. ******* Do you suffer from diabetes mellitus?
   - Yes
   - No
2. ******* Do you suffer from haematological diseases (*e.g.*, anaemia, thrombocytopenia, thrombocythemia, polyglobulia, thalassaemia, monoclonal gammopathy, etc.)?
   - Yes
   - No
3. ******* If you answered “yes” to the previous question, please state below which ones:

___________________________________________________________

_____________________________________________________________________

1. ******* Do you suffer or have you suffered from cancer (including lymphoma) in the past? *
   - Yes
   - No
2. ******* If you answered “yes” to the previous question, please state below which ones:

___________________________________________________________

_____________________________________________________________________

1. ******* Do you suffer from a respiratory disease (*e.g.*, asthma, chronic bronchitis, emphysema, infections, bronchiectasis, etc.)?
   - Yes
   - No
2. ******* If you answered “yes” to the previous question, please state below which ones:

___________________________________________________________

_____________________________________________________________________

1. ******* Do you suffer or have you suffered from a psychiatric condition (*e.g.*, anxiety, depression, bipolar disorder, personality disorder, schizophrenia, etc.)?
   - Yes
   - No
2. ******* If you answered “yes” to the previous question, please state below which ones:

___________________________________________________________

_____________________________________________________________________

1. ******* Do you suffer or have you suffered from any neurological pathology (*e.g.*, stroke, migraine, Parkinson's disease, infectious diseases of the brain, etc.)?
   - Yes
   - No
2. ******* If you answered “yes” to the previous question, please state below which ones:

___________________________________________________________

_____________________________________________________________________
