# Supplementary material for: Comorbidity in myasthenia gravis: multicentric, hospital-based, and controlled study of 178 Italian patients
Source: Neurol Sci. 2024 Feb 22;45(7):3481–94. doi: 10.1007/s10072-024-07368-0 (PMC11176220; doi:10.1007/s10072-024-07368-0)
Supplement: Supplementary file 2 — Supplementary file2 (DOCX 28 KB) [file 10072_2024_7368_MOESM2_ESM.docx]

**- Supplementary File 2 -**

Table comparing the prevalence of each comorbidity belonging to a specific category between subjects with Myasthenia Gravis and Healthy Controls.

|  | **Myasthenia Gravis**  **N=178** | **Healthy Controls**  **N=178** | ***p*** |
| --- | --- | --- | --- |
| Thyroid disease | 13 (7.3) | 17 (9.6) | 0.4 |
| Thyroid nodules | 8 (4.5) | 14 (7.9) | 0.2 |
| Goitre | 5 (2.8) | 3 (1.7) | 0.7 |
| Kidney disease | 13 (7.3) | 12 (6.7) | 0.8 |
| Kidney stones | 8 (4.5) | 8 (4.5) | 1 |
| Renal cyst | 1 (0.6) | 1 (0.6) | 1 |
| Chronic renal disease | 3 (1.7) | 3 (1.7) | 1 |
| Cardiovascular diseases | 41, (238) | 21, (11.8) | 0.005 |
| Ischemic cardiopathy | 12 (6.7) | 7 (3.9) | 0.24 |
| Arrythmia | 11 (6.2) | 8 (4.5) | 0.48 |
| Ventricular hypertrophy | 5 (2.8) | 0 | 0.06 |
| Cardiac failure | 4 (2.2) | 0 | 0.12 |
| Valvulopathies | 9 (5.1) | 4 (2.2) | 0.16 |
| Atherosclerosis | 6 (3.4) | 0 | 0.03 |
| Aneurysm | 2 (1.1) | 0 | 0.5 |
| Ocular disease | 14 (7.9) | 23 (12.9) | 0.1 |
| Cataract | 7 (3.9) | 16 (9) | 0.05 |
| Glaucoma | 4 (2.2) | 4 (2.2) | 1 |
| Retinopathy | 3 (1.7) | 3 (1.7) | 1 |
| Gastrointestinal disease | 24, (13.5) | 41, (23) | 0.02 |
| GERD | 11 (6.2) | 32 (18) | 0.001 |
| Gallstones | 6 (3.4) | 0 | 0.03 |
| Gastritis | 6 (3.4) | 9 (5.1) | 0.43 |
| Liver disease | 5 (2.8) | 0 | 0.06 |
| Hematological disease | 17, (9.6) | 8, (4.5) | 0.06 |
| Anaemia | 9 (5.1) | 7 (3.9) | 0.6 |
| Other cytopenia | 3 (1.7) | 0 | 0.3 |
| MGUS | 3 (1.7) | 1 (0.6) | 0.6 |
| Hypogammaglobulinemia | 2 (1.1) | 0 | 0.5 |
| Coagulopathies | 1 (0.6) | 0 | 1 |
| Non-Thymic tumours | 28, (15.7) | 16, (9) | 0.05 |
| Uterine | 5 (2.8) | 1 (0.6) | 0.2 |
| Breast | 2 (1.1) | 1 (0.6) | 1 |
| Prostate | 2 (1.1) | 0 | 0.5 |
| Urinary | 2 (1.1) | 4 (2.2) | 0.7 |
| Cutaneous | 3 (1.7) | 1 (0.6) | 0.6 |
| Respiratory | 3 (1.7) | 1 (0.6) | 0.6 |
| Gastrointestinal | 2, (1.1) | 1, (0.6) | 1 |
| CNS | 4 (2.2) | 0 | 0.1 |
| Glands | 6, (3.4) | 1, (0.6) | 0.1 |
| Lymphoma | 1, (0.6) | 0 | 1 |
| Unknown | 0 | 7, (3.9) | 0.02 |
| Respiratory disease | 23 (12.9) | 6 (3.4) | 0.001 |
| COPD | 10 (5.6) | 1 (0.6) | 0.006 |
| Asthma | 8 (4.5) | 3 (1.7) | 0.13 |
| OSAS | 7, (3.9) | 1, (0.6) | 0.07 |
| Interstitial lung disease | 1, (0.6) | 1, (0.6) | 1 |
| Neurological disease | 59, (33.1) | 18, (10.1) | <0.0001 |
| History of Stroke/TIA | 16, (9) | 5, (2.8) | 0.01 |
| Migraine | 14, (7.9) | 6, (3.4) | 0.07 |
| Epilepsy | 6, (3.4) | 0 | 0.03 |
| PD and parkinsonism | 6, (3.4) | 1, (0.6) | 0.1 |
| Neuropathy | 18, (10.1) | 5, (2.8) | 0.005 |
| Motor neuron disease | 3, (1.7) | 0 | 0.3 |
| Fibromyalgia | 2, (1.1) | 2, (1.1) | 1 |
| Radiculopathy | 5, (2.8) | 0 | 0.06 |
| Psychiatric disease | 27, (15.2%) | 27, (15.2%) | 1 |
| Anxiety | 16, (9) | 17, (9.6) | 0.9 |
| Depression | 16, (9) | 10, (5.6) | 0.2 |
| Psychosis | 2, (1.1) | 2, (1.1) | 1 |
| Autoimmune comorbidity, n, % | 44, (24.7) | 21, (11.8) | 0.002 |
| Hashimoto’s thyroiditis | 24, (13.5) | 12, (6.7) | 0.04 |
| Graves’ disease | 2, (1.1) | 2, (1.1) | 1 |
| Rheumatoid arthritis | 2, (1.1) | 2, (1.1) | 1 |
| IBD | 5, (2.8) | 2, (1.1) | 0.5 |
| Psoriasis | 4, (2.2) | 3, (1.7) | 1 |
| Connectivitis | 7, (3.9) | 1, (0.6) | 0.07 |
| CIDP | 1, (0.6) | 0 | 1 |
| Vasculitis | 1, (0.6) | 0 | 1 |
| Polymyalgia | 1, (0.6) | 0 | 1 |
| Stiffman syndrome | 1, (0.6) | 0 | 1 |
| Two or more autoimmune | 6, (3.4) | 1, (0.6) | 0.1 |

Abbreviations: GERD, gastroesophageal reflux disease; MGUS; monoclonal gammopathy of undetermined significance; COPD, chronic obstructive pulmonary diseases; OSAS, obstructive sleep apnoea syndrome; TIA, transient ischemic attack; PD, Parkinson’s disease; IBD, inflammatory bowel diseases; CIDP, chronic inflammatory polyneuropathy.
